# Gen Z’s Willingness to Adopt Plant-Based Diets: Empirical Evidence from Greece, India, and the UK

**DOI:** 10.3390/foods13132076

**Published:** 2024-06-30

**Authors:** Elena Raptou, Amalia Tsiami, Giulia Negro, Veena Ghuriani, Pooja Baweja, Slim Smaoui, Theodoros Varzakas

**Affiliations:** 1 Department of Agricultural Development, Democritus University of Thrace, 68200 Orestiada, Greece; elenra@agro.duth.gr; 2 London Geller College of Hospitality and Tourism, University of West London, London W5 5RF, UK; amalia.tsiami@uwl.ac.uk (A.T.); 21314658@student.uwl.ac.uk (G.N.); 3 Department of Computer Science, Maitreyi College, University of Delhi, Delhi 110021, India; vghuriani@maitreyi.du.ac.in; 4 Department of Botany, Maitreyi College, University of Delhi, Delhi 110021, India; pbaweja@maitreyi.du.ac.in; 5 Laboratory of Microbial and Enzymes Biotechnology and Biomolecules (LMEBB), Centre of Biotechnology of Sfax (CBS), University of Sfax-Tunisia, Road of Sidi Mansour Km 6, P.O. Box 1177, Sfax 3018, Tunisia; slim.smaoui@cbs.rnrt.tn; 6 Department Food Science and Technology, University of the Peloponnese, 24100 Kalamata, Greece

**Keywords:** attitudes toward plant-based foods, willingness to adopt plant-based diets, meal preparation involvement, ordered probit model analysis, PCA, cluster analysis

## Abstract

Comprising the largest population cohort on this planet, Gen Z presents a future-oriented consumer segment driven by climate change and food. This study sought to investigate Gen Z’s perceptions toward plant-based foods and diets and explore the relationship that attitude components, meal preparation involvement, personal and lifestyle factors, and perceived barriers in adopting a plant-based diet have with willingness to adopt green-eating practices. Using cross-sectional data from university students in Greece, India, and the UK, various tools were employed to determine the factors influencing youths’ consumer behavior toward animal-protein substitutes. PCA indicated the underlying dimensions of students’ viewpoints on plant-based foods, whereas hierarchical and k-means clustering provided the cluster structure. An ordered probit model was estimated to delineate Gen Z’s willingness to adopt plant-based diets and distinguish among mostly unwilling, somewhat willing, and mostly willing youths. Our findings identified two consumer segments, namely proponents and opponents of plant-based foods and diets, with statistically significant differences in the perceived health benefits of plant-based diets, attachment to animal-based proteins, perceived exclusion of animal-based foods, dissatisfaction with plant-based foods’ attributes, and demand for ensuring adequate protein intake. The ordered probit model estimates showed that there is a “homogeneity” in the factors influencing youths’ intention to adopt plant-based diets, with attitude components, meal preparation indicators, perceived barriers to eating “green”, and personal factors, such as self-assessed knowledge of healthy eating and physical activity, being strongly associated with students’ willingness to switch to plant-based diets in all three countries. Mapping potential obstacles and enablers in terms of shifting to more green-eating behaviors, our findings could add information to better understand the factors affecting food choice and youths’ transition to a more sustainable lifestyle.

## 1. Introduction

In the modern food systems, there is a noticeable consumer shift to animal-protein substitutes and more plant-based dietary patterns motivated by health and environmental concerns, as well as contemporary ethical debates [1,2,3]. The alternative protein market is booming and it is expected to increase from EUR 1.5 billion in 2018 to EUR 2.4 billion in 2025, whereas the gradual replacement of meat with its plant-based substitutes is perceived as a “win-win” situation encompassing positive health and environmental aftereffects [4,5]. A great body of the literature has stated numerous health repercussions of meat, and especially processed meat, consumption, such as type 2 diabetes, life expectancy decrease, cardiovascular diseases, and even cancer [6,7,8,9]. In addition, meat consumption has been linked with a substantial environmental burden, including greenhouse gas emissions, water pollution, deforestation, and biodiversity loss [7,10].

Thus, reducing meat consumption and replacing it with plant-based substitutes has an essential role in human health improvement and the mitigation of environmental damage from livestock farming and food production. Nutrition and health interventions promoting plant-based alternatives are therefore indispensable for ensuring population wellbeing and an ecologically sustainable future, whereas a shift of dietary choice to plant-based protein could also guarantee nutritional adequacy, food security and food sufficiency [4]. However, a great proportion of consumers still seem to be reluctant toward plant-based foods, despite their well-documented positive outcomes. Although there is an increase in the number of people switching to a plant-based diet, meat consumption remains highly ingrained in Western culture, and the disposition to reduce the consumption of animal products seems to be relatively low [11]. Recent literature has explored the meat-rich Western dietary pattern [11,12] indicating a social pressure to decrease meat consumption [13]. This reveals the necessity for further research on the potential barriers in switching to plant-based diets and choosing plant-protein alternatives over animal-based protein. Orienting consumer demand toward reduced meat consumption and increasingly plant-based diets will likely be a vigorous challenge for strengthening environmental and health policy.

An investigation of consumer attitudes toward plant-based diets is considered critical since it can uncover underlying motivational and structural factors influencing the acceptance level of plant-based foods and contribute to the design of nutrition strategies and the promotion of eating “green”. Many factors have been found to affect consumers’ willingness to adopt a more plant-based diet and reduce their meat consumption, including meat attachment factors [2,12,14,15], food choice motives [15,16,17,18], and perceptions toward plant-based diets [2,16,18,19]. Furthermore, a significant body of the literature noted that perceived health benefits and environmental concerns also had a major influence in establishing plant-based food consumption patterns [3,5,15,20,21,22].

Today, Generation Z (Gen Z) comprises a dynamic consumer bracket making food choices determined by a variety of biological (i.e., hunger, appetite, taste), economic (i.e., income, price, availability), social (i.e., culture, family, peers, food trends), psychological (i.e., mood, stress, food neophobia), and lifestyle (e.g., culinary skills, information) factors, perceptions, and attitudes [23]. Growing up in a universal foodie culture with a broad range of food options, those in Gen Z have developed plentiful senses of taste, differentiating them from previous generations. The eating habits of those in Gen Z are linked with environmental and health concerns, which have a major role in establishing food preferences and choices [24,25]. Employing individual-level data from a sample of university students in Minnesota, Pelletier et al. [26] noted that positive attitudes for foods from local, organic and/or sustainable sources were common in youths exhibiting higher dietary quality. In addition, Marinova and Bogueva [23] underlined that Gen Z’s food choices are mostly based on attitude, information, and care, instead of cultural factors and the hedonic value of food. Using cross-sectional data from Greece, Kamenidou et al. [27] highlighted the role of meat substitutes in traditional Mediterranean gastronomy (e.g., “fakorizo”—lentils with rice), although it seemed that those in Gen Z are unaware of the available plant-based alternatives to meat and dairy. Recent evidence from the UK showed that there is a significant rise in the consumption of plant-based foods, especially in adults 24 to 39 years old with higher income, and supported the hypothesis that plant-based products may help the shift to more green-eating practices [28].

The Gen Z cohort has a crucial role in the future of food, since youths demand change, create new food trends, shape future food production, and opt for more resilient food systems. The present study investigated Gen Z’s attitudes and perceptions toward animal-protein substitutes, and also explored youths’ willingness to adopt plant-based diets in Greece, India, and the UK. The empirical analysis employed individual-level data from a sample of university students, and first sought to determine the underlying dimensions of their attitudes toward plant-based foods and define youth segments characteristics with respect to their level of awareness toward green-eating patterns and plant-based protein. Furthermore, the factors influencing youths’ willingness to adopt plant-based diets were explored in terms of meal preparation involvement, personal and lifestyle factors, attitude components, and perceived barriers to adopting plant-based diets after controlling for various sociodemographic characteristics.

## 2. Materials and Methods

### 2.1. Sampling Selection and Measures

The present research employed cross sectional data from a sample of Gen Z (generation Z) university students in three countries, namely Greece, India, and the UK. Participants were adults born between the mid-to-late 1990s and the early 2010s of any sexual orientation, gender identity, race, or ethnic background. The research received ethical approval from the University of West London (UWL/REC/SHT-00845). The quantitative data selection tool was a validated formal questionnaire, appropriately adapted to the nutrition interests and lifestyles of Gen Z [29]. After the pre-test of the adapted questionnaire, the final version was distributed online to university students from the UK, Greece, and India. Participants were recruited via email announcements including the link to the online form of the questionnaire and a consent to research participation form, ensuring personal data confidentiality. Researchers used snowball nonprobability convenience sampling to enhance inclusion of a broader audience [30]. Participation was voluntary and respondents did not receive any form of compensation for taking part in the research. The questionnaire completion time was estimated to be approximately 15 min. Finally, 528 valid questionnaires were selected (107 questionnaires from UK, 115 from Greece, and 306 from India). The time period varied since it occurred between April and September 2022 for the UK, September–November 2023 for India, and October 2023–January 2024 for Greece.

Students’ responses were coded for ease of interpretation in the subsequent analysis. In the first part of the questionnaire, contextual information was included to describe participants’ sociodemographic characteristics. Respondents were asked to declare their gender identification with a categorical variable taking a value of 0 for “male”, a value of 1 for “female”, and a value of 2 for “other”. Since responses were classified in the first two categories, the gender indicator was finally expressed as a dummy variable. The age indicator was also recoded as a dichotomous variable taking a value of 0 for “18–23 years old” and a value of 1 for “over 23 years old”. Educational attainment was described through two categories, namely undergraduate (taking a value of 1 for undergraduates and 0 otherwise) and postgraduate level (taking a value of 1 for postgraduates and 0 otherwise). In addition, students’ term-time residence was explored through a set of three dummies, corresponding to “living with parents or relative”, “university accommodation”, and “living in a private accommodation (either alone or with other students/friends)”.

Meal preparation involvement was evaluated through a set of four dichotomous indicators expressing students’ responsibility for food shopping (value of 1 for “most/all responsibility”, value of 0 for “otherwise”), meal planning (value of 1 for “most/all”, value of 0 for “otherwise”), and cooking (value of 1 for “most/all”, value of 0 for “otherwise”), as well as a self-assessment of culinary skills (value of 1 for “very good/excellent culinary skills”, value of 0 for “otherwise”). Individuals’ knowledgeability of healthy eating was measured through a 5-point scale ranging from 1 to 5 (1: having no knowledge, 5: having excellent knowledge). To facilitate subsequent econometric analysis, the 5-point rating scale was recoded into a dichotomous indicator, in which responses ranging from 1 to 3 in the 5-point scale were recoded as 0, reflecting “less knowledgeable” participants, whereas responses ranging from 4 to 5 were recoded as 1, corresponding to “highly knowledgeable” students. Dichotomous indicators were also constructed to express additional individual personal and lifestyle factors in terms of healthiness of current dietary habits, activity level, and media influence. In particular, a self-perceived balanced diet was described by a dummy variable taking a value of 1 for students defining their current diet as healthy/balanced, and a value of 0 otherwise. Students were also asked to describe their activity level, and hence a dummy was constructed taking a value of 1 for highly active participants and a value of 0 for less active individuals. Students were also asked to state whether social media and TV advertising influence people to buy plant-based foods. A dichotomous indicator was created with a value of 1 in the case of a positive response and 0 otherwise.

**Figure 1:**
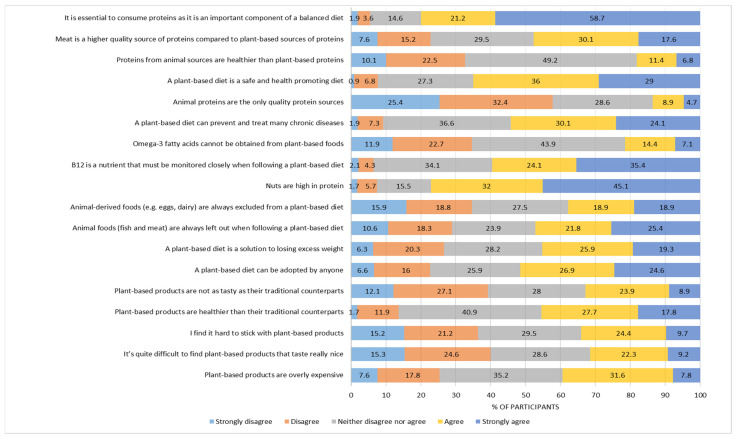
Students’ attitudes toward plant-based diets and foods (% of participants).

To explore students’ viewpoints toward the perceived barriers to adopting a plant-based diet, a set of four dichotomous indicators was constructed to delineate an absence of interest in consuming plant proteins, perceived difficulty in following a plant-based diet, lack of knowledge about the quality of plant proteins, and lack of knowledge about the health benefits of consuming plant proteins. In the last part of the questionnaire, an 18-item variable rated on a five-point Likert scale (totally disagree/disagree/neither disagree nor agree/agree/totally agree) was employed to investigate students’ attitudes toward plant-based foods and diets. This scale is analytically presented in Figure 1. Finally, an individual’s willingness to adopt a plant-based dietary routine was expressed through an ordinal variable taking a value of 0 for mostly unwilling respondents, a value of 1 for somewhat willing respondents, and a value of 2 for mostly willing participants.

### 2.2. Methods of Analysis

The present study sought to investigate students’ attitudes toward plant-based foods/diets, outline their consumption profile, and explore individuals’ endorsements of green-eating patterns in Greece, India, and the UK. Cross-sectional data were analyzed through various statistical techniques to define the main components that underline individuals’ perceptions toward plant-based products and delineate different consumer segments. Econometric analysis also estimated the factors influencing willingness to adopt plant-based diets in Greek, Indian, and English students. The models employed are analytically presented below.

(i) Factor analysis

In order to reveal the underlying components depicting students’ attitudes toward plant-based diets and foods, an exploratory factor analysis (EFA) was performed, which comprises a widely used multivariate technique for the investigation of scale validity and the reduction in the dimensionality in a set of variables. The output of the EFA can be also adopted to facilitate further statistical analysis [31,32,33,34]. In the present study, EFA was employed to test the potential interdependencies among the observed variables of the 18-item scale describing individuals’ attitudes toward plant-based diets and food products and uncover the underlying theoretical constructs referred as latent variables [35]. The EFA was conducted via principal component analysis (PCA) with the varimax rotation method to maximize the sum of the variance of the square loadings [36,37]. The factors produced by the EFA application were enrolled in subsequent statistical analysis [38,39].

Several prerequisites were suggested before proceeding to the EFA. In particular, the Kaiser–Meyer–Olkin (KMO) criterion of sampling adequacy and the Bartlett’s test of sphericity were adopted to assess the suitability of sample sizes for factor analysis and the fitness of the data [35,40,41]. KMO estimations over the value of 0.50 indicate the data adequacy of the factorial model [42], whereas the Bartlett’s test of sphericity tests the null hypothesis that the correlation matrix coincides with the identity matrix, assessing its factorability [43]. The selection of the number of factors was based on the K1 rule, so the extracting factors with eigenvalues over 1 were retained, while the rest were discarded without losing much of the original variability [44]. To achieve the optimal factor solution, all variables with estimated factor loadings over 0.40 on more than one factor were removed from the analysis [45]. Cronbach’s α coefficients were also calculated to indicate the level of reliability and unidimensionality of the set of variables constructing each factor. For values greater than 0.50, all constructs were considered to be in the acceptable range [46,47,48,49].

(ii) Cluster Analysis

Cluster analysis (CA) was employed for grouping university students into similar segments based on their attitudes and perceptions toward plant-based diets and foods. The data set included the factors produced by the preceding EFA application. The clusters were considered as mutually exclusive with internal homogeneity and intragroup heterogeneity [50]. In a recent review study, Oyewole and Thopil [51] analytically presented and discussed the algorithms and strategies of CA. In the context of the present research, the CA was performed through both hierarchical and non-hierarchical (k-means) clustering techniques. First, hierarchical cluster analysis was applied to uncover the number of clusters after classifying cases into homogenous groups by aggregating them, one at a time, in a series of sequential steps [52,53,54,55,56]. For hierarchical clustering, Ward’s method was enrolled as the grouping technique, whereas the squared Euclidean distance was adopted as a measure of similarity between cases. The dendrogram produced by Ward’s method was used to determine the number of clusters. Then, the k-means algorithm was used to develop the clustering model, setting a priori the number of clusters that resulted from Ward’s method [57]. To further validate the cluster structure obtained by CA application, discriminant analysis was employed to explore the accuracy of the cluster solution [58,59].

(iii) Econometric approach—the ordered probit model

To address students’ endorsement of green-eating patterns, a theoretical framework was employed based on the assumption that individual’s willingness to adopt plant-based diets is related to sociodemographic factors, meal preparation indicators, personal and lifestyle characteristics, attitudes toward plant-based diets and foods, and perceived barriers toward the adoption of green-eating behaviors. Since students’ level of willingness as a dependent variable has possible responses of a discrete and ordinal nature, the empirical analysis adopted an ordered probit model to explore individuals’ responsiveness to plant-based eating patterns. The conceptual basis of this approach is rooted in the field of random utility maximization [60] and has been elaborated in the literature to model ordinal survey responses [61,62,63,64,65]. Therefore, by leveraging the random utility modelling approach, it is assumed that for the individual, the latent dependent variable $y_{i}^{*}$ (individual’s endorsement of green-eating) incorporates two elements: first, a linear combination of a vector of independent variables *x*_i_ and the parameter vector *β* that have to be estimated reflecting the relationship between $y_{i}^{*}$ and the variables in *x_i_*; and second, an unobserved random variable *ε*_i_, assumed to be independent and identically distributed with a standard normal distribution that is $\varepsilon_{i}\sim Ν\left(0,1\right)$ reflecting unobserved factors of the alternatives for the individual i (i = 1, 2, 3…N) [66,67]:

(1) $$y_{i}^{*}=x_{i}^{\prime }\beta +\varepsilon_{i}$$

Since the dependent variable in the former equation is unobserved, it is measured by a set of ordered responses through a censoring mechanism. In that case, the utility of each alternative is assumed to fall within a specific utility interval. Therefore, the observed ordered dependent variable *y*_i_ reflecting individuals’ alleged level of willingness to adopt plant-based diets is expressed as follows:

(2) $$\begin{array}{l} y_{i}=0\;if\;y_{i}^{*}\leq \mu_{0}(=0)\\ y_{i}=1\;if\;\mu_{0}<y_{i}^{*}\leq \mu_{1}\\ y_{i}=2\;if\;\mu_{1}<y_{i}^{*}\leq \mu_{2}\\ y_{i}=J\;if\;\mu_{J-1}<y_{i}^{*} \end{array}$$

where μs are threshold parameters to be estimated in order to indicate the range of the normal distribution associated with specific values of the stated response variable $y_{i}^{*}$ along with the parameter vector *β*, subject to the constraint *μ*_0_ < *μ*_1_ < … < *μ*_*j*−1_. For reasons of identification, one restriction is imposed on the threshold parameters, that is, *μ*_0_ = 0. The probability associated with the observed outcome *j*, where *j =* 0, 1, 2,…, *J*, can be specified by the following:

(3) $$Prob\left(y_{i}=0\right)=\Phi \left(-x_{i}^{\prime }\beta \right)\;Prob\left(y_{i}=1\right)=\Phi \left(\mu_{1}-x_{i}^{\prime }\beta \right)-\Phi \left(-x_{i}^{\prime }\beta \right)\;Prob\left(y_{i}=2\right)=\Phi \left(\mu_{2}-x_{i}^{\prime }\beta \right)-\Phi \left(\mu_{1}-x_{i}^{\prime }\beta \right)\;Prob\left(y_{i}=J\right)=1-\Phi \left(\mu_{J-1}-x_{i}^{\prime }\beta \right)$$

where Φ(.) is the standard normal cumulative density function.

The parameters of the model are estimated by maximizing the log-likelihood function. Therefore, the likelihood function and the log-likelihood function can be expressed as follows:

(4) $$L=\prod_{i=1}^{N}\prod_{j=0}^{J}{\left\{\Phi \left(\mu_{j}-x_{i}^{\prime }\beta \right)-\Phi \left(\mu_{j-1}-x_{i}^{\prime }\beta \right)\right\}}^{y_{ij}}$$

(5) $$LogL=\sum_{i=1}^{N}\sum_{j=0}^{J}y_{ij}log\left\{\Phi \left(\mu_{j}-x_{i}^{\prime }\beta \right)-\Phi \left(\mu_{j-1}-x_{i}^{\prime }\beta \right)\right\}$$

where *y_ij_* is a dichotomous indicator, taking a value of 1 in the case that the ith individual presents the level of willingness j and 0 otherwise.

In the context of the present research, the ordinal dependent variable took the following possible values: mostly unwilling to adopt plant-based diets (*y* = 0), somewhat willing to adopt plant-based diets (*y* = 1), and mostly willing to adopt plant-based diets (*y* = 2). Furthermore, the vector of explanatory variables encompassed the sociodemographic dummies (gender, age, educational attainment, accommodation), the meal preparation indicators (responsibility for food shopping, responsibility for meal planning, responsibility for cooking, very good/excellent culinary skills), the personal and lifestyle indicators (knowledgeable about healthy eating, self-perceived balanced diet, high activity level, social media and TV ad influence), the factors resulted from the EFA application, and the indicators describing the perceived barriers in adopting a plant-based diet (absence of interest in consuming plant proteins, perceived difficulty in following a plant-based diet, lack of knowledge about the quality of plant protein, lack of knowledge about the health benefits of consuming plant proteins).

The interpretation of the estimated coefficients of the ordered probit model can be ambiguous, especially for the intermediate categories, because the changes in the probabilities associated with the intermediate categories cannot be signed a priori [67]. Thus, the calculation of the marginal effects is necessary when the ordered probit model is employed. In the context of the present study, the marginal effects of variables on the probability of an individual having each of the three possible levels of willingness can by calculated from the estimated coefficients by the following expressions:

(6) $$\frac{\partial Prob\left[y_{i}=0\right]}{\partial x_{i}}=-\phi \left(x_{i}^{\prime }\beta \right)\beta \;\frac{\partial Prob\left[y_{i}=1\right]}{\partial x_{i}}=\left[\phi \left(-x_{i}^{\prime }\beta \right)-\phi \left(\mu -x_{i}^{\prime }\beta \right)\right]\beta \;\frac{\partial Prob\left[y_{i\;}=2\right]}{\partial x_{i}}=\phi \left(\mu -x_{i}^{\prime }\beta \right)\beta$$

where *φ*(.) is the density function of the univariate normal distribution. The marginal effect for a continuous variable indicates the change in the predicted probability resulting from an incremental unit change in the dependent variable. For a dummy variable, the marginal effect represents the difference between the two probabilities with and without the variable. All variables are held at their mean levels except for the variable being interpreted.

## 3. Results and Discussion

It is crucial to decode the motives behind Gen Z’s food choices in order to capture the factors associated with the dietary transition to more plant-based food options. Understanding why consumers choose to abstain from meat products, and instead turn to more “green” eating practices, may help health and nutrition scholars to design and implement effective policy regulations that will improve individuals’ dietary patterns. Factors related to environmental protection, food attributes, and health improvement should be explored to clarify their influence on eating choices [68,69,70,71,72] and consumers’ adherence to more sustainable diets [73].

### 3.1. Students’ Profile in Greece, India, and the UK

Table 1 provides an analytical description of the sample. Of the 528 participants, 21.7% and 58% were Greek and Indian university students, respectively, whereas the remaining 20.3% corresponded to their English counterparts. The predictors presented a wide range of variability across the three countries of interest. The application of chi-square tests revealed that the country of residence had a strong relationship with sociodemographic characteristics, meal preparation indicators, personal and lifestyle factors, perceived barriers to adopting a plant-based diet, and willingness to shift toward green dietary patterns (Table 1). In total, the great majority of participants were female undergraduates, 18–23 years old, living in a private accommodation with their parents/relatives or with friends/flatmates. In terms of gender, the sample of English students was close to the distribution in the national population, whereas the male group was underrepresented in both Greece and India. Recent literature has noted a constant gender effect in survey participation, with women being more responsive compared to men, especially in online research designs [74,75,76]. To avoid biased estimates in subsequent statistical analysis, a weighting scheme was applied based on respondents’ gender in the Greek and Indian subsamples (each group was given a weight corresponding to the proportion in the total population) [77].

Furthermore, most of the university students in the UK and a great proportion of Greeks were themselves mainly responsible for food shopping (92.5% and 75.7%, respectively), and for planning (84.1% and 66.1%, respectively) and preparing their own meals (92.5% and 68.7%, respectively) compared to students in India. English and Greek students’ high involvement with meal preparation may be probably due to the lower rates of cohabitation with parents or other relatives. Recent evidence showed that university students who had to move away from their parents’ home and live alone for the first time established independence, formed new dietary habits, and became responsible for the preparation of their meals [78,79], although longitudinal studies suggested that the healthiness of eating practices declined in most cases [80].

Knowledge about healthy eating was statistically related to the country classification indicator, with English students considering themselves more informed on healthy dietary patterns than their Indian and Greek counterparts (chi-square test = 10.02, *p* < 0.01). Similarly, English students seemed quite convinced that they follow a balanced diet (71%), whereas Greek and Indian students were less confident in their dietary choices (64.3% and 57.2%, respectively) (chi-square test = 6.901, *p* < 0.05). Statistically significant differences regarding the inhibiting factors in following a green diet were also observed across student groups. In particular, the absence of interest in consuming plant proteins and perceived difficulty were reported as the main barriers to adopting a plant-based diet, mostly by English students. In addition, a statistically significant association between the country of residence and the willingness to adopt a more plant-based diet was revealed (chi-square test = 56.092, *p* < 0.01), with Indian students being more receptive to shifting toward a more plant-based diet (46.5%) compared to their Greek and English counterparts (14.4% and 19.6%, respectively) (Table 1).

### 3.2. Students’ Attitudes toward Plant-Based Diets and Foods

Participants’ attitudes toward plant-based diets and foods are described on Figure 1. A high proportion of the students seemed to perceive plant-based foods as overly expensive products (39.4%) of inferior taste compared to conventional products (32.8%), although they shared the opinion that some of them may have a really nice taste (39.9%). The taste of a product is a crucial sensory aspect that has a major effect on consumers’ acceptance [81]. Previous research also indicated that the perceived poor sensory offering of meat substitutes prevented consumers from incorporating them into their diet [82], suggesting that the fear of disliking their flavor usually discourages their adoption [83,84]. Regarding the alleged difficulty of including plant foods in one’s eating routine, a great proportion of respondents supported the notion that they are much healthier than their traditional counterparts and agreed that a plant-based diet can be easily adopted by anyone (45.5% and 51.5%, respectively). Furthermore, the majority of the students noted that plant-based diets are safe and health-promoting (65%), and considered them particularly effective in the prevention and the treatment of chronic diseases (54.2%) as well as in weight loss (45.2%). To further support previous research linking plant-based diets with health benefits [3,12,83,85,86,87], our findings also showed that young adults attribute significant health-related improvements to the adoption of “green” eating practices.

However, perceptions that plant-based foods were more expensive than conventional products may restrict their uptake and discourage individuals from including them in their diets, since price constitutes a determinant of critical importance in dietary choices and food consumption [88,89,90]. Widespread beliefs that meat products are less expensive, whereas their plant-based substitutes are perceived as higher-priced food options [21,91,92], may constitute a barrier toward product trial [22,93]. However, recent evidence showed that consumers who follow plant-based diets report lower food expenditures than omnivorous individuals [72]. Therefore, in order to promote plant-based dietary practices and encourage their adoption, nutrition interventions should also consider economic repercussions in addition to environmental and health consequences. This is especially important for consumer groups, such as young adults and university students, as they are at a transitional stage signifying major lifestyle changes. Many of them had to move away from their parents’ homes, live by their own, and manage their financial affairs. Therefore, such consumer groups might be more price-sensitive, and hence they should be informed that a shifting to a more plant-based diet will not destabilize their economic situation.

In addition, participants’ responses showed that significant proportions of students were convinced that animal foods (like fish) and animal-derived foods (like eggs and dairy products) are excluded from green-eating patterns, revealing a lack of information on the different types of plant-based diets (e.g., vegan vs. lacto-vegan) in Gen Z. With respect to protein consumption, approximately 80% of respondents declared that proteins comprise an important component of a balanced diet, whereas 59.5% of the total sample added that B12 should be monitored when following a plant-based diet. Although great proportions of participants agreed that animal proteins are not the only quality protein sources (57.8%), seeing nuts as an excellent protein source (77.1%), almost half of the respondents considered meat as a higher-quality source of proteins compared to plant-based protein sources (47.7%) (Figure 1). Students’ viewpoints on the protein adequacy of plant-based foods indicated insufficient information on alternative protein sources in Gen Z, and might reflect consumers’ mistrust of the quality of plant-based protein. However, recent food research has shown that a balanced plant-based diet with a combination of various amino acids can ensure protein intake adequate to cover the human body’s needs [94]. Furthermore, new food products, such as plant-based products with highly bioavailable plant proteins (e.g., soy), and new microbial fermentation techniques can also ensure protein adequacy in plant-based diets [95]. Health and nutrition campaigns could help young adults better understand their dietary and food options, and hence increase their awareness of plant-based dietary practices.

In order to assess the dimensionality of the components delineating students’ attitudes toward plant-based diets and foods, explorative factor analysis (EFA) was employed through principal component analysis (PCA) with varimax rotation. The KMO measure was 0.796 (>0.50), implying data adequacy for the PCA [35,40,41], whereas the Bartlett’s test of sphericity indicated variable correlation and suitability for structure detection (chi-square = 2327.589, *p* < 0.01). The EFA of the 18 variables extracted a five-factor solution (eigenvalue > 1), and the total variance explained was 58.281%. The Cronbach’s α coefficients were greater than 0.5, showing that all the constructs were in the acceptable range [46,47,48,49].

Table 2 presents the EFA and the reliability analysis output on participants’ perceptions toward plant-based diets and foods. The first factor, labeled as “health benefits of plant-based diets”, included five variables explaining 13.440% of the total variance and had a reliability coefficient of 0.744. This factor loaded attributes related to the healthiness and safety of plant-based diets and foods and the adaptation to plant-eating behavior. The second factor accounted for 13.321% of the total variance and comprised 4 out of the 18 variables that mainly described students’ views on plant-based food products’ characteristics, such as taste and price. This second factor was entitled as “dissatisfaction with plant-based food attributes” and had a reliability coefficient of 0.743. Furthermore, the third factor was found to explain 12.204% of the total variance, and was labeled as “ensuring adequate protein intake in plant-based diets” according to the content of the three variables included. Reliability analysis provided a Cronbach α measure equal to 0.651. The fourth factor, “perceived exclusion of animal-based foods”, incorporated three variables explaining 10.129% of the total variance with a reliability coefficient of 0.656. This factor involved attributes regarding students’ concerns about the consumption restrictions on products of animal origin, such as fish, meat, eggs, and dairy foods, in plant-based diets. The fifth factor, “attachment to meat proteins”, incorporated three variables explaining 9.276% of the total variance, with a reliability coefficient of 0.556. This factor involved attributes regarding students’ perceptions toward the quality of plant-based proteins (Table 2).

### 3.3. Students’ Segments According to Attitudes toward Plant-Based Diets

In order to identify student segments on the basis of their attitudes toward plant-based diets and food options, cluster analysis was employed based on the factors obtained from the PCA (Table 3).

First, Ward’s hierarchical clustering method was applied to define the optimal number of clusters, followed by k-means algorithm for stabilization purposes [57]. To enhance the validity of our findings, we further explored different cluster models ranging from a two-cluster solution to a five-cluster solution via discriminant analysis [59]. The classification results showed that the two-cluster model provided the best interpretive cluster solution, since it presented higher percentages of the original grouped cases correctly classified (97.2%) compared to the remaining alternatives. T-tests for the equality of means indicated statistically significant differences between the two clusters in terms of perceptions toward health benefits of plant-based diets, dissatisfaction with plant-based food attributes, ensuring adequate protein intake in plant-based diets, perceived exclusion of animal-based foods, and attachment to animal-based proteins. To fulfill the analytical description of the students’ profile on the basis of their behavior toward plant-based foods and diets, cross-tabulation and Pearson’s χ^2^ statistics were also calculated to identify statistically significant differences between clusters (Table 4).

Cluster 1, labeled as the “plant-based diet proponents”, accounted for 53.8% of the sample and presented the lowest mean factor scores compared to the other segment in “attachment to animal based proteins” (2.536 vs. 3.198, t-test = −10.571, *p* < 0.001), “perceived exclusion of animal-based foods” (2.467 vs. 3.772, t-test = −21.730, *p* < 0.001), and “dissatisfaction with plant-based food attributes” (2.487 vs. 3.500, t-test = −16.497, *p* < 0.001). The students of this segment seemed to trust the quality of the plant-based protein sources, while being highly satisfied with the attributes of the foods included and well-informed on the food options in plant-based dietary patterns (Table 3). Cluster 2 was labeled as “plant-based diet opponents” and included 46.2% of respondents. The students classified in this cluster were more skeptical about the health benefits of plant-based foods compared to the members of the other segment (3.443 vs. 3.660, t-test = 3.370, *p* < 0.01), and also questioned the adequacy of protein intake in plant-based eating habits (4.183 vs. 4.033, t-test = −2.318, *p* < 0.05) (Table 3).

Table 4 presents the sociodemographics, the meal preparation habits, and the personal/lifestyle characteristics of each cluster. Non-parametric tests showed that sociodemographics are irrelevant to cluster membership. On the other hand, 68% of the proponents of plant-based diets perceived their eating patterns as balanced compared to 54.10% of the opponents’ group (chi-square test = 10.652, *p* < 0.01) Furthermore, the responsibility for food shopping and meal planning were found to be statistically associated with cluster profile. Thus, 52.80% of plant-based diet proponents organize their food purchases by themselves compared to 40.60% of plant-based diet opponents (chi-square test = 7.984, *p* < 0.01), whereas 50% of the students classified in Cluster 1 schedule their meals compared to 37.5% of the opponents’ segment (chi-square test = 10.995, *p* < 0.01). Most of the times, individuals who get involved in meal preparation are more interested in nutrition information, new food products, and novel food technologies. They are also more likely to read information on food packaging, seek information from various domains, such as social media, cook forums, and food influencers, and they become more familiar with the new food products on the supermarket shelves. As a consequence, an increase in familiarity with plant-based foods may decrease food neophobia reactions and overcome motivational adoption barriers to a plant-based dietary shift [96,97,98]. According to Table 4, involvement with meal preparation activities can also increase individuals’ experiences with foods, which in turn may limit neophobic behaviors [97,99].

### 3.4. Willingness to Adopt a Plant-Based Diet

An ordered probit regression was adopted to model the willingness to adopt a more plant-based diet and investigate the influence of sociodemographic characteristics, meal preparation indicators, personal/lifestyle factors, and perceived barriers to following plant-based dietary patterns among mostly unwilling, somewhat willing, and mostly willing consumers. Table 5, Table 6 and Table 7 present the ordered probit model estimates and the calculated marginal effects for the student subsamples (Greek, Indian, English). Concerning the ordered probit model procedure for the English student subsample, the Likelihood Ratio test indicated that the explanatory variables employed in the estimation process are appropriate (LR chi-squared test = 133.66, *p* < 0.01). Similarly, the Wald chi-squared tests indicated that the set of the explanatory variables employed in the ordered probit regression for the Greek and Indian student subsamples are significant (Wald chi-squared = 47.13, *p* < 0.01, Wald chi-squared = 90.75, *p* < 0.01, respectively).

#### 3.4.1. The Role of Sociodemographics

With respect to sociodemographic characteristics, several regressors were found to be statistically significant. Term-time residence, and particularly living alone or with friends, constituted a determinant factor in Greek students’ decisions to adopt plant-based diets (*β* = 1.014, *p* < 0.01). Thus, individuals who declared living alone or with friends in a private accommodation were less likely by 28.7% to reject plant-based diets and had an increased likelihood by 19.2% and 9.4% to be somewhat willing or mostly willing to adopt more green-eating behaviors, respectively (Table 5). It seems that moving away from the family home has a substantial influence on plant-based diet acceptance, especially in Greek youths. The process of food choice and eating is profoundly linked with social norms and rituals in Greek families. There are strong cultural connections between meat consumption and Sunday family gatherings and dinners, whereas meat dishes are considered typical in special religious or national events [97,100]. In addition, the immediate social environment has a critical role on dietary patterns and food choices. Family environments usually establish specific eating rules, forming their members’ dietary habits and influencing taste preferences and food enjoyment. Most of the times, individuals follow the informal dietary guidelines set in their household, and find obstacles to eating “green” if other family members and relatives are unwilling to adopt a more plant-based diet [101].

Furthermore, gender (*β* = 0.982, *p* < 0.05) and educational attainment (*β* = 2.480, *p* < 0.01) were positively related to willingness to adopt plant-based behaviors in the Indian subsample (Table 6). Female students were less likely by 20.8% to be somewhat willing and more likely by 33.1% to be mostly willing to adjust to more plant-based eating patterns. Postgraduates also had an increased likelihood of adopting plant-based diets by 57.5% and a lower probability by 3.1% and 54.5% to be adverse to or slightly interested in following green-eating patterns, respectively. Our findings further support previous research indicating gender differences in meat endorsement, with men being more reluctant to reduce or avoid meat consumption compared to women, whereas the latter were more open to vegetarianism and plant-based diets [3,15,102]. Cultural dimensions of masculinity preserve stereotypes that consuming meat is masculine, resulting in lower acceptance of vegetarianism [103].

#### 3.4.2. The Role of Meal Preparation Activities

Involvement with meal preparation activities was found to be strongly related to engagement in plant-based diets in all subsamples. Responsibility for cooking was inversely related to the willingness to adopt more plant-based diets in Greeks (*β* = −1.232, *p* < 0.01). The estimated marginal effects revealed that students who prepared and cooked their own meals were more likely by 26.5% to restrict themselves from the adoption of more plant-based diets (Table 5). Recent evidence indicated that family dietary habits continue to have a critical role in university students’ cooking choices and habits, even after they have moved out on their own [104,105]. Therefore, students whose families attributed major importance to meat dishes were rather more positive about meat consumption and favored meatless meals less. Although the Mediterranean diet comprises the prevalent dietary pattern in Greece and it is a mainly plant-based diet, a great body of the recent literature noted a substantial decrease in Mediterranean diet adherence in the Mediterranean Basin, mostly due to lifestyle and socioeconomic changes and a shift to Western-type dietary patterns [106,107,108]. In addition, university life constitutes a transitional stage for young adults who have to compromise between health concerns and convenience. The subsequent prioritization of convenience over health may result in an increased consumption of ready-to-eat dishes, which in most cases list meat, fish, and/or dairy products in their ingredients and have a high fat content [109].

On the contrary, responsibility for meal planning was found to be positively associated with adherence to plant-based diets in English students (*β* = 1.321, *p* < 0.01), with individuals engaging in meal planning tasks having a 43.9% lower probability of being unwilling to adopt green-eating patterns (Table 7). Furthermore, culinary skills were found to be negatively related to willingness to adopt plant-based diets in both Indian (*β* = −0.862, *p* < 0.05) and English subsamples (−1.087, *p* < 0.05). Thus, Indian students with self-assessed excellent culinary skills had a lower likelihood of adopting more plant-based dietary practices by 29.9% (Table 6), whereas their English counterparts were more likely to be unwilling to move toward green-eating habits by 29.6% (Table 7). As Feher et al. [3] underlined, there is limited information on plant-based dishes and their preparation. At the same time, individuals lack knowledge about the types of products that could substitute animal-based protein, as those ingredients are often hard to find in food stores [110,111] and are mostly perceived as premium products of a higher price [22]. Furthermore, the widespread perception that the preparation of plant-based meals is more demanding and time-consuming may discourage the adoption of plant-based dietary patterns [94,112,113]. Information obtained through various domains (i.e., mass media, social media) on plant-based meal recipes and the cooking process could motivate young adults to experiment with new ingredients and also improve their cooking skills. Information on the health and environmental benefits of plant-based eating habits could also motivate Gen Z to consume more ethically and overcome the perceived price obstacle of plant-based foods.

#### 3.4.3. The Role of Personal and Lifestyle Factors

Several personal and lifestyle factors were found to determine willingness to adopt plant-based diets. In particular, self-assessed knowledge about healthy eating was positively linked with both Greek and English students’ intentions to adopt green-eating patterns (Greeks: *β* = 0.759, *p* < 0.05, English: *β* = 0.856, *p* < 0.05) (Table 5 and Table 7). More information can result in rising familiarity with green-eating dietary patterns and a higher self-efficacy in replacing food products like meat with its substitutes [15]. Thus, higher self-assessed knowledge levels would allow Gen Z consumers to become more prone to the endorsement of animal-protein substitutes, whereas their repeated exposure to plant-based meals could help increase positive appraisals and reduce neophobic reactions over time [15,96,99].

With regard to physical activity, high levels were inversely associated with willingness to adopt plant-based diets in Indian students (*β* = −0.755, *p* < 0.01). Thus, individuals with a strong commitment in physical exercise were less likely by 27.8% to move toward more plant-based dietary patterns (Table 6). On the contrary, physically active Greek students were less likely by 14.3% to be opposed to the adoption of plant-based diets (*β* = 0.710, *p* < 0.10) (Table 5). Differences on the direction of physical activity’s influence on the acceptability of plant-based diets between Greeks and Indians may be attributed to their established eating habits.

As explained above, although the Mediterranean diet is supposed to comprise the most prevalent dietary pattern in Greece, during the last decades there has been a noticeable shift to more Westernized dietary habits, especially in the youth population [114,115,116]. Young adults engaged in systematic physical exercise may be more aware of health issues and nutrition patterns with subsequent health benefits. Thus, they may become less hesitant about trying new food products, such as plant-based foods, or adopt eating practices that will help them improve health outcomes and wellbeing [21]. On the other hand, India presents the highest percentage of vegetarians worldwide, with 29% of the population over 15 years old following a vegetarian diet [117]. However, as Singh et al. [118] noted, there is a substantial increase in obesity and overweight rates, marking a nutrition transition away from “faith vegetarianism”, mostly attributed to changes in eating and cooking habits, such as the replacement of whole plant foods with refined carbohydrates and processed and energy-dense fried foods. Therefore, this increase in negative health outcomes may have made physically active Indian youths question the quality of the plant-based dietary patterns, although this should be interpreted with caution and investigated further.

#### 3.4.4. The Role of Attitudes toward Plant-Based Diets

As expected, attitudes toward plant-based diets were significantly associated with the willingness to adopt green-eating patterns (Table 5, Table 6 and Table 7). Thus, consumers’ awareness of ensuring protein adequacy was found to be positively related to the willingness to adopt plant-based diets in all subsamples. The greatest marginal effect, estimated in the English subsample, showed that individuals with a higher level of awareness were less likely by 30.9% to restrict themselves from adopting more plant-based diets. Furthermore, a strong attachment to animal-based proteins seemed to restrain consumers from switching to more plant-based dietary practices in both Greek (*β* = −0.475, *p* < 0.10) and Indian students (*β* = −0.218, *p* < 0.10). The perceived exclusion of animal-based foods was inversely linked with intentions to follow a more plant-based diet in both Indian (*β* = −0.278, *p* < 0.05) and English students (*β* = −1.982, *p* < 0.01). The greatest marginal effects, estimated in the English subsample, showed that individuals supporting the idea that certain food options, such as fish, eggs, and dairy products, are excluded from plant-based diets have a higher probability by approximately 50% to be mostly unwilling to adopt them. Dissatisfaction with plant-based food attributes was a disincentive to adopting more plant-based dietary choices in both Greek (*β* = −0.538, *p* < 0.10) and Indian students (*β* = −0.540, *p* < 0.01).

On the other hand, perceived health benefits were positively related to the willingness to adopt more plant-based diets in Indian participants (*β* = 0.406, *p* < 0.01). Surprisingly, English students who agreed on the health benefits of plant-based diets had a higher probability by 40.3% to restrict themselves from the transition to eating “green”. This finding should be taken into consideration with caution, since there may exist external factors, such as the price of plant-based foods, which could limit the purchasing power of English consumers and undermine their shift to more green-eating patterns. With respect to the perceived barriers in adopting plant-based diets, several indicators were found to be statistically significant (Table 5, Table 6 and Table 7). The perceived difficulty of following a plant-based diet was negatively associated with switching to more green-eating patterns in Indian students (*β* = −0.748, *p* < 0.01). In addition, the lack of knowledge about the health benefits of consuming plant proteins had the major influence on willingness to adopt plant-based diets, with English students suggesting that lack of information may hamper individuals’ transitions to plant-based choices.

Our findings corroborated previous research indicating that motivational factors, such as perceived health benefits and wellbeing improvement [3,4,12,15,21,22], constitute facilitators of the transition to more plant-based diets, whereas dissatisfaction with attributes, such as taste [3,22,23,81,83] and price [21,88,89,90], discourages their adoption. As discussed above, familiarity and convenience with plant-protein substitutes could also influence consumers’ willingness to adopt plant-based diets. Due to lack of experience in terms of purchasing, cooking, and preparing meals, animal-protein substitutes might be perceived as inconvenient [81,93]. Furthermore, Kerslake et al. [22] noted that omnivores (higher familiarity with meat products) were reluctant to try meat substitutes and perceived meat products as more convenient, whereas vegans and vegetarians (low familiarity with meat products) considered the consumption of meat substitutes to be convenient. Convenience and familiarity seem to be interrelated, since both may constitute barriers toward the adoption of plant-based diets. Plant-based foods comprise a novel category, and neophobic reactions may restrain consumers from their trial [96,97,98]. In addition, the traditional belief that meat comprises a main component of a balanced meal can further deter individuals from consuming plant-based meat substitutes [96] and also preserve established beliefs on the dietary superiority of the animal protein, enhancing consumers’ mistrust toward the quality of the plant-based protein and the protein adequacy of plant-based eating patterns.

### 3.5. Study Limitations

Due to the nature of the sample selection method (snowball sampling), there was an overrepresentation of specific sociodemographic segments (i.e., women, undergraduate students) and homogeneity especially in meal preparation involvement, such as food shopping, meal planning, and cooking. In particular, the great majority of English students were found to systematically engage in meal preparation and be responsible for food shopping, meal planning, and cooking. A random sampling selection design in future research could ensure the participation of more sociodemographic groups (i.e., employed young adults, those in Gen Z with lower educational status) with differences in lifestyle, eating patterns, nutrition interests, and motivational factors toward the adoption of plant-based diets. Furthermore, a focus on sociodemographics related to the upbringing and family background of those in Gen Z could reveal crucial information on the mechanisms behind potential changes in eating behavior and food preferences. A longitudinal research design could also provide insights on how attitudes and purchasing behavior toward plant-based foods change over time. Given that the present study explored perceptions and attitudes related with the perceived healthiness of the plant-based diets and protein adequacy, the quality and the attributes of the plant-based foods, and meat attachment, future research should take into consideration the environmental aspects and explore the potential interrelations between them.

### 3.6. Implications for the Food Industry

The present research has offered a holistic approach to addressing the linkage of both motivational (i.e., dimensions of attitudes) and structural adoption barriers (e.g., difficulty in following a plant-based diet) with those in Gen Z’s intention to shift to more green-eating practices. It has underlined the factors associated with youths’ awareness of eating “green” (proponent vs. opponent) and willingness to adopt more plant-based diets, and has noted the critical role of meal preparation involvement (responsibility for food shopping, meal planning, cooking, culinary skills) and attitudes toward plant-based diets and plant-based food options. It also explored how personal factors and perceived barriers were related to the transition to plant-based diets, and revealed the mechanism linking health benefits, dissatisfaction with plant-based food attributes, protein adequacy of animal food substitutes, and perceived exclusion of animal-based foods with intention to eat “green”, as well as the inverse relationship between meat protein endorsement (meat attachment) and willingness to shift to plant-based eating patterns. Our findings showed that these factors had a major influence on Gen Z’s purchasing behavior in all three counties, indicating that beyond cultural differences, there is a certain “homogeneity” in young adults, perhaps due to mass media/social media penetration and influence on youths’ lifestyles. To better communicate animal-protein substitutes to the public, health and nutrition scholars should consider how consumers perceive the associated health impacts and the environmental burden of meat production. Also, from a business perspective, it is of great importance to unveil young people’s decision making process in order to cover “future” consumers’ needs and also create stable, long-lasting and “environmentally friendly” choices.

Past evidence showed that individuals seemed uncertain about the quality of the plant-protein sources [94,119,120,121]. Information on plant-based food options could help increase familiarity and also counter neophobic reactions toward plant-based products. To increase consumers’ acceptability toward animal-protein substitutes and reduce neophobia, recent evidence highlighted the need for product development in such a way to make “novel foods resemble familiar foods” [97]. This could also improve their sensory content and satisfy consumers’ preferences for better taste and texture. Furthermore, in order to satisfy young adults’ demand for protein and nutrient adequacy, food industry should also invest in the fortification of the plant-based foods to supply individuals with all the necessary nutrients for a balanced diet (i.e., proteins, iron, B12). Despite the “healthiness challenge”, food fortification could contribute to reaching sustainability goals, since the subsequent decrease in meat production might help moderate the environmental costs. Previous research noted that the combination of both health and environmental goals could help marketers promote plant-based food options with efficacy, since health and environmental benefits are usually integrated in consumers’ conscience [5,97,122]. Beyond product improvement, marketers should also pay attention to the disruption in the market of “green” products due to the inadequate labeling regulation. Alcorta et al. [94] commented on the existing initiatives and labeling regulations, noting that the heterogeneity of regulations across countries can “create a climate of uncertainty and result in a stifling of investment and innovation”. Using clear and creative labeling could help better promote plant-based foods and increase their acceptance in young adults.

## 4. Concluding Remarks

To conclude, plant-based dietary patterns have grabbed the interest of consumers, scientists, and policy makers for their potential to integrate healthiness and sustainability. There are also emerging market opportunities for the food industry to meet an increasing demand for healthy and “environmentally friendly” food products and practices. Although the market of animal-protein substitutes is continually expanding, there are still issues that should be considered and improved to curb the structural and motivational barriers toward the acceptance of plant-based foods, enhance Gen Z’s shift to eating “green”, and provide incentives to the food industry for research and innovation.

## Figures and Tables

**Table 1:** Sample description (N = 528).

Variable		Total Sample (N = 528)	Greek Students (N_1_ = 115)	Indian Students (N_2_ = 306)	English Students (N_3_ = 107)	Chi-Square Test	*p*-Value
***Gender***	Male	77	19	2	56	170.443	<0.001
		(14.6)	(16.5)	(0.7)	(52.3)		
	Female	451	96	304	51		
		(85.4)	(83.5)	(99.3)	(47.7)		
***Age***	18–23	397	100	294	3	380.646	<0.001
		(75.2)	(87.0)	(96.1)	(2.8)		
	>23	131	15	12	104		
		(24.8)	(13.0)	(3.9)	(97.2)		
***Educational attainment***	Undergraduates	473	110	298	65	119.844	<0.001
		(89.6)	(95.7)	(97.4)	(60.7)		
	Postgraduates	55	5	8	42		
		(10.4)	(4.3)	(2.6)	(39.3)		
***Accommodation***	Living with parents/relative	197	22	162	13	77.174	<0.001
		(37.3)	(19.1)	(52.9)	(12.1)		
	University accommodation	56	9	24	23	16.783	<0.001
		(10.6)	(7.8)	(7.8)	(21.5)		
	Living in a private accommodation	252	80	105	67	53.559	<0.001
		(47.7)	(69.6)	(34.3)	(62.6)		
***Meal preparation***	Responsibility for food shopping	249	87	63	99	212.525	<0.001
		(47.2)	(75.7)	(20.6)	(92.5)		
	Responsibility for meal planning	229	76	63	90	161.142	<0.001
		(43.4)	(66.1)	(20.6)	(84.1)		
	Responsibility for cooking	241	79	63	99	196.84	<0.001
		(45.6)	(68.7)	(20.6)	(92.5)		
	Very good/excellent culinary skills	67	10	14	43	92.864	<0.001
		(12.7)	(8.7)	(4.6)	(40.2)		
***Personal and lifestyle factors***	Knowledgeable about healthy eating	262	65	134	63	10.02	0.007
		(49.6)	(56.5)	(43.8)	(58.9)		
	Self-perceived balanced diet	325	74	175	76	6.901	0.032
		(61.6)	(64.3)	(57.2)	(71.0)		
	High activity level	83	20	47	16	0.320	0.852
		(15.7)	(17.4)	(15.4)	(15.0)		
	Social media and TV ad influence	179	40	93	46	5.666	0.059
		(33.9)	(34.8)	(30.4)	(43.0)		
***Perceived barriers in adopting a plant-based diet***	Absence of interest in consuming plant proteins	108	14	50	44	57.064	<0.001
		(20.5)	(12.2)	(16.7)	(52.0)		
	Perceived difficulty in following a plant-based diet	113	38	29	46	85.048	<0.001
		(21.4)	(33.0)	(9.0)	(54.0)		
	Lack of knowledge about the quality of plant proteins	74	19	39	16	2.195	0.334
		(14.0)	(16.0)	(13.0)	(19.0)		
	Lack of knowledge about the health benefits of consuming plant proteins	83	21	53	9	2.595	0.273
		(15.7)	(18.0)	(17.0)	(10.0)		
***Willingness to adopt a plant-based diet***	Mostly unwilling	63	20	16	27	56.092	<0.001
		(14.8)	(18.0)	(7.0)	(27.8)		
	Somewhat willing	226	75	100	51		
		(53.2)	(67.0)	(46.0)	(52.6)		
	Mostly willing	136	16	101	19		
		(32.0)	(14.4)	(46.5)	(19.6)		

% percentages are in parentheses.

**Table 2:** Factor analysis (PCA) and reliability analysis output on students’ attitudes toward plant-based foods (N = 528).

Eigenvalue	Total Variance Explained %	Factors	Factor Loading	Mean	S.D. *	Cronbach α
		***Factor 1: health benefits of plant-based diets***				
		Plant-based products are healthier than their traditional counterparts	0.750	3.479	0.974	
		A plant-based diet is a solution to losing excess weight	0.739	3.318	1.177	
2.419	13.440	A plant-based diet is a safe and health-promoting diet	0.680	3.852	0.949	0.744
		A plant-based diet can be adopted by anyone	0.584	3.472	1.205	
		A plant-based diet can prevent and treat many chronic diseases	0.540	3.676	0.970	
		***Factor 2: dissatisfaction with plant-based food attributes***				
		It’s quite difficult to find plant-based products that taste really nice	0.763	2.852	1.195	
2.381	13.231	I find it hard to stick with plant-based products	0.754	2.922	1.202	0.743
		Plant-based products are not as tasty as their traditional counterparts	0.735	2.903	1.159	
		Plant-based products are overly expensive	0.616	3.142	1.044	
		***Factor 3: Ensuring adequate protein intake in plant-based diets***				
		B12 is a nutrient that must be monitored closely when following a plant-based diet	0.750	3.864	1.020	
2.197	12.204	It is essential to consume proteins as it is an important component of a balanced diet	0.704	4.313	0.975	0.651
		Nuts are high in protein	0.695	4.131	0.986	
		***Factor 4: perceived exclusion of animal-based foods***				
		Animal foods (fish and meat) are always left out when following a plant-based diet	0.841	3.330	1.317	
1.823	10.129	Animal-derived foods (e.g., eggs, dairy, eggs) are always excluded from a plant-based diet	0.827	3.063	1.331	0.656
		Omega-3 fatty acids cannot be obtained from plant-based foods	0.535	2.818	1.048	
		***Factor 5: attachment to meat proteins***				
		Proteins from animal sources are healthier than plant-based proteins	0.790	2.824	0.992	
1..670	9.276	Meat is a higher quality source of proteins compared to plant-based sources of proteins	0.636	3.350	1.158	0.556
		Animal proteins are the only quality protein sources	0.628	2.352	1.096	

* S.D.: standard deviation.

**Table 3:** Cluster analysis results for students’ attitudes toward plant-based diets (N = 528).

Variables	Total Sample		Cluster 1 Plant-Based Diet Proponents (53.8%)		Cluster 2 Plant-Based Diet Opponents (46.2%)		Levene’s Test for Equality of Variances		t-Test for Equality of Means	
	Mean	S.D. *	Mean	S.D.*	Mean	S.D. *	F-Test	*p*-Value	t-Test	*p*-Value
Health benefits of plant-based diets	3.560	0.746	3.660	0.756	3.443	0.717	0.691	0.406	3.370	0.001
Dissatisfaction with plant-based food attributes	2.955	0.866	2.487	0.716	3.500	0.689	1.278	0.259	−16.497	<0.001
Ensuring adequate protein intake in plant-based diets	4.102	0.763	4.033	0.860	4.183	0.624	27.373	<0.001	−2.318	0.021
Perceived exclusion of animal-based foods	3.070	0.953	2.467	0.749	3.772	0.630	7.389	0.007	−21.730	<0.001
Attachment to animal-based proteins	2.842	0.789	2.536	0.709	3.198	0.727	0.141	0.708	−10.571	<0.001

* S.D.: standard deviation.

**Table 4:** Cluster profile (N = 528).

Variable	Cluster 1 Plant-Based Diet Proponents (53.8%)	Cluster 2 Plant-Based Diet Opponents (46.2%)	Chi-Square Test *	*p*-Value
Gender (female)	249	202	2.519	0.113
	87.70%	82.80%		
Age (>23)	210	187	0.511	0.457
	73.90%	76.60%		
Education (postgraduates)	31	24	0.164	0.686
	10.90%	9.80%		
Living with parents/relatives	96	101	3.233	0.072
	33.80%	41.40%		
University accommodation	32	24	0.284	0.594
	11.30%	9.80%		
Living in a private accommodation	143	109	1.697	0.193
	50.40%	44.70%		
Responsible for food shopping	150	99	7.984	0.005
	52.80%	40.60%		
Responsible for meal planning	142	87	10.995	0.001
	50.00%	35.70%		
Responsible for cooking	139	102	2.697	0.101
	48.90%	41.80%		
Very good/excellent culinary skills	33	34	0.635	0.426
	11.60%	13.90%		
Knowledgeable about healthy eating	151	111	3.094	0.079
	53.20%	45.50%		
Self-perceived balanced diet	193	132	10.652	0.001
	68.00%	54.10%		
High activity level	51	32	2.323	0.127
	18.00%	13.10%		
Social media and TV ad influence	89	90	1.802	0.179
	31.30%	36.90%		

* α = 5%.

**Table 5:** Willingness to adopt a plant-based diet—ordered probit model results for Greek students.

Variables	Ordered Probit Model Estimates	Marginal Effects		
		Mostly Unwilling	Somewhat Willing	Mostly Willing
***Sociodemographic characteristics***				
Gender (female)	0.442	−0.109	0.059	0.050
	(0.307)	(0.081)	(0.052)	(0.038)
Age (>23)	−0.154	0.037	−0.018	−0.019
	(0.413)	(0.092)	(0.040)	(0.054)
Education (postgraduates)	0.372	−0.079	0.027	0.053
	(0.816)	(0.143)	(0.023)	(0.148)
University accommodation	−0.150	0.040	−0.025	−0.015
	(0.630)	(0.178)	(0.122)	(0.056)
Living in a private accommodation	1.014 ***	−0.287 ***	0.192 **	0.094 **
	(0.350)	(0.108)	(0.092)	(0.043)
***Meal preparation***				
Responsible for food shopping	−0.746	0.156 *	−0.046	−0.110
	(0.539)	(0.089)	(0.049)	(0.108)
Responsible for meal planning	0.616	−0.163	0.100	0.063
	(0.406)	(0.116)	(0.085)	(0.043)
Responsible for cooking	−1.232 ***	0.265 ***	−0.080	−0.185 **
	(0.337)	(0.079)	(0.070)	(0.074)
Very good/excellent culinary skills	0.402	−0.086	0.029	0.057
	(0.501)	(0.090)	(0.025)	(0.087)
***Personal/lifestyle factors***				
Knowledgeable about healthy eating	0.759 **	−0.205 *	0.129	0.076 **
	(0.374)	(0.106)	(0.088)	(0.035)
Self-perceived balanced diet	−0.524	0.120 *	−0.053	−0.068
	(0.348)	(0.073)	(0.039)	(0.051)
High activity level	0.710 *	−0.143 **	0.033	0.110
	(0.423)	(0.068)	(0.050)	(0.092)
Social media and TV ad influence	−0.276	0.072	−0.044	−0.028
	(0.270)	(0.075)	(0.050)	(0.028)
***Attitudes toward plant-based diets***				
Health benefits of plant-based diets	0.159	−0.040	0.022	0.018
	(0.222)	(0.056)	(0.034)	(0.023)
Dissatisfaction with plant-based food attributes	−0.538 *	0.135 *	−0.076	−0.059
	(0.319)	(0.081)	(0.056)	(0.038)
Ensuring adequate protein intake in plant-based diets	0.545 ***	−0.137 ***	0.077 *	0.060 **
	(0.198)	(0.049)	(0.040)	(0.030)
Perceived exclusion of animal-based foods	0.093	−0.023	0.013	0.010
	(0.196)	(0.049)	(0.028)	(0.022)
Attachment to animal-based proteins	−0.475 *	0.119 *	−0.067	−0.052 *
	(0.249)	(0.068)	(0.049)	(0.031)
***Perceived barriers in adopting a plant-based diet***				
Absence of interest in consuming plant proteins	0.746 *	−0.142 **	0.018	0.123
	(0.458)	(0.066)	(0.065)	(0.110)
Perceived difficulty in following a plant-based diet	0.648	−0.146 *	0.059	0.086
	(0.434)	(0.085)	(0.040)	(0.077)
Lack of knowledge about the quality of plant proteins	−0.524	0.153	−0.109	−0.044
	(0.524)	(0.172)	(0.147)	(0.032)
Lack of knowledge about the health benefits of consuming plant proteins	−0.315	0.088	−0.059	−0.029
	(0.417)	(0.128)	(0.098)	(0.032)
*μ*_1_	−1.057			
	(1.496)			
*μ*_2_	1.510			
	(1.504)			
**Log Likelihood**	**−74.085**			
**Wald chi-squared(22) = 47.13, *p*-value = 0.001**				

* statistical significance at *p* < 0.10, ** statistical significance at *p* < 0.05, *** statistical significance at *p* < 0.01. Standard errors are in parentheses, accommodation (living with parents/relative): omitted variable.

**Table 6:** Willingness to adopt a more plant-based diet—ordered probit model results for Indian students.

Variables	Ordered Probit Model Estimates	Marginal Effects		
		Mostly Unwilling	Somewhat Willing	Mostly Willing
***Sociodemographic characteristics***				
Gender (female)	0.982 **	−0.122	−0.208 ***	0.331 ***
	(0.476)	(0.097)	(0.043)	(0.119)
Age (>23)	0.224	−0.015	−0.073	0.088
	(0.441)	(0.035)	(0.134)	(0.169)
Education (postgraduates)	2.480 ***	−0.031 ***	−0.545 ***	0.575 ***
	(0.746)	(0.011)	(0.049)	(0.047)
University accommodation	−0.362	0.027	0.113	−0.139
	(0.304)	(0.030)	(0.083)	(0.112)
Living in a private accommodation	−0.214	0.012	0.072	−0.085
	(0.206)	(0.013)	(0.068)	(0.081)
***Meal preparation***				
Responsible for food shopping	−0.121	0.007	0.041	−0.048
	(0.383)	(0.024)	(0.126)	(0.150)
Responsible for meal planning	0.290	−0.013	−0.102	0.115
	(0.312)	(0.013)	(0.111)	(0.123)
Responsible for cooking	−0.006	0.000	0.002	−0.002
	(0.296)	(0.016)	(0.101)	(0.117)
Very good/excellent culinary skills	−0.862 **	0.098	0.201 ***	−0.299 ***
	(0.403)	(0.073)	(0.048)	(0.109)
***Personal/lifestyle factors***				
Knowledgeable about healthy eating	0.190	−0.010	−0.065	0.075
	(0.202)	(0.011)	(0.069)	(0.080)
Self-perceived balanced diet	0.011	−0.001	−0.004	0.004
	(0.196)	(0.011)	(0.067)	(0.078)
High good activity level	−0.755 ***	0.070 *	0.207 ***	−0.277 ***
	(0.273)	(0.042)	(0.055)	(0.088)
Social media and TV ad influence	0.309	−0.015	−0.108	0.123
	(0.199)	(0.010)	(0.071)	(0.078)
***Attitudes toward plant-based diets***				
Health benefits of plant-based diets	0.406 ***	−0.022 **	−0.139 ***	0.161 ***
	(0.138)	(0.010)	(0.049)	(0.055)
Dissatisfaction with plant-based food attributes	−0.540 ***	0.029 ***	0.185 ***	−0.215 ***
	(0.149)	(0.010)	(0.055)	(0.059)
Ensuring adequate protein intake in plant-based diets	0.266 **	−0.014 *	−0.091 *	0.106 **
	(0.134)	(0.008)	(0.047)	(0.053)
Perceived exclusion of animal-based foods	−0.278 **	0.015 **	0.095 **	−0.110 **
	(0.109)	(0.007)	(0.039)	(0.044)
Attachment to animal-based proteins	−0.218 *	0.012	0.075*	−0.087 *
	(0.121)	(0.008)	(0.042)	(0.048)
***Perceived barriers in adopting a plant-based diet***				
Absence of interest in consuming plant proteins	−0.158	0.009	0.053	−0.062
	(0.263)	(0.018)	(0.086)	(0.103)
Perceived difficulty in following a plant-based diet	−0.748 **	0.072	0.200 ***	−0.272 **
	(0.353)	(0.059)	(0.059)	(0.109)
Lack of knowledge about the quality of plant proteins	0.406	−0.017 *	−0.144	0.161
	(0.270)	(0.010)	(0.097)	(0.104)
Lack of knowledge about the health benefits of consuming plant proteins	0.308	−0.014	−0.108	0.122
	(0.240)	(0.011)	(0.085)	(0.095)
*μ*_1_	1.361			
	(0.919)			
*μ*_2_	0.729			
	(0.924)			
**LogLikelihood**	**−137.626**			
**Wald chi-squared** **(22) = 90.75, *p*-value < 0.001**				

* statistical significance at *p* < 0.10, ** statistical significance at *p* < 0.05, *** statistical significance at *p* < 0.01. Standard errors are in parentheses, accommodation (living with parents/relative): omitted variable.

**Table 7:** Willingness to adopt a more plant-based diet—ordered probit model results for English students.

Variables	Ordered Probit Model Estimates	Marginal Effects		
		Mostly Unwilling	Somewhat Willing	Mostly Willing
***Sociodemographic variables***				
Gender (female)	−0.691	0.185	−0.173	−0.012
	(0.426)	(0.121)	(0.115)	(0.015)
Age (>23)	−0.172	0.046	−0.044	−0.003
	(1.080)	(0.313)	(0.299)	(0.014)
Education (postgraduates)	0.631	−0.146	0.130	0.016
	(0.465)	(0.099)	(0.088)	(0.020)
University accommodation	0.886	−0.171	0.136	0.035 **
	(0.659)	(0.106)	(0.068)	(0.053)
Living in a private accommodation	0.405	−0.108	0.101	0.007
	(0.569)	(0.157)	(0.149)	(0.011)
***Meal preparation***				
Responsible for food shopping	−0.962	0.153 *	−0.100 *	−0.053
	(0.887)	(0.084)	(0.053)	(0.096)
Responsible for meal planning	1.321 **	−0.439 **	0.427 *	0.012
	(0.635)	(0.225)	(0.224)	(0.015)
Responsible for cooking	0.211	−0.058	0.054	0.003
	(0.945)	(0.276)	(0.265)	(0.012)
Very good/ excellent culinary skills	−1.087 **	0.296 **	−0.275 **	−0.021
	(0.481)	(0.135)	(0.130)	(0.022)
***Personal/lifestyle factors***				
Knowledgeable about healthy eating	0.856 *	−0.225 *	0.209 *	0.016
	(0.454)	(0.128)	(0.119)	(0.020)
Self-perceived balanced diet	−0.280	0.068	−0.062	−0.006
	(0.448)	(0.106)	(0.095)	(0.013)
High activity level	0.408	−0.087	0.076	0.012
	(0.716)	(0.132)	(0.103)	(0.032)
Social media and TV ad influence	0.014	−0.004	0.003	0.000
	(0.424)	(0.107)	(0.099)	(0.008)
***Attitudes toward plant-based diets***				
Health benefits of plant-based diets	−1.603 ***	0.403 ***	−0.372 ***	−0.031
	(0.557)	(0.146)	(0.141)	(0.034)
Dissatisfaction with plant-based food attributes	−0.261	0.066	−0.061	−0.005
	(0.322)	(0.082)	(0.075)	(0.008)
Ensuring adequate protein intake in plant-based diets	1.231 *	−0.309 *	0.286 *	0.023
	(0.662)	(0.170)	(0.162)	(0.027)
Perceived exclusion of animal-based foods	−1.982 ***	0.498 ***	−0.461 ***	−0.038
	(0.526)	(0.126)	(0.132)	(0.040)
Attachment to animal-based proteins	−0.711	0.179	−0.165	−0.014
	(0.474)	(0.128)	(0.119)	(0.018)
***Perceived barriers in adopting a plant-based diet***				
Absence of interest in consuming plant proteins	−0.246	0.061	−0.056	−0.005
	(0.469)	(0.113)	(0.104)	(0.010)
Perceived difficulty in following a plant-based diet	0.655	−0.171	0.159	0.012
	(0.486)	(0.133)	(0.124)	(0.016)
Lack of knowledge about the quality of plant proteins	2.097 ***	−0.253 ***	0.005	0.248
	(0.764)	(0.084)	(0.186)	(0.211)
Lack of knowledge about the health benefits of consuming plant proteins	−2.262 **	0.742 ***	−0.730 ***	−0.012
	(0.913)	(0.197)	(0.201)	(0.014)
*μ*_1_	−8.737			
	(3.889)			
*μ*_2_	−5.310			
	(3.734)			
**LogLikelihood**	**−38.940**			
**LR chi-squared (22) = 73.21, *p*-value < 0.001**				

* statistical significance at *p* < 0.10, ** statistical significance at *p* < 0.05, *** statistical significance at *p* < 0.01. Standard errors are in parentheses, accommodation (living with parents/relative): omitted variable.

## Data Availability

The data selected for this study cannot be made publicly available. The ethics approval does not include consent for the availability of the datasets that support the conclusions of this manuscript.
